# Case Report: Scurvy—a modifiable cause of psychiatric refractoriness and lithium pharmacokinetic abnormalities in autism

**DOI:** 10.3389/fpsyt.2025.1741734

**Published:** 2026-01-23

**Authors:** Marie Geiser, Nicolas Guggisberg, Josephine Convertini, Severine Crettol, Nicolas Ansermot, Vincent Guinchat

**Affiliations:** 1 Département de Psychiatrie, Centre Hospitalier Universitaire Vaudois (CHUV), Lausanne, Switzerland; 2 Ecole de médecine, Université de Lausanne, Lausanne, Vaud, Switzerland; 3 Département de Médecine interne, Centre Hospitalier Universitaire Vaudois (CHUV), Lausanne, Switzerland

**Keywords:** autism, case report, challenging behaviors, intellectual disability, lithium, scurvy, vitamin C deficiency

## Abstract

Scurvy, although rare in developed countries, can notably occur in individuals with extreme dietary selectivity, particularly those with neurodevelopmental disorders. We describe the case of an 18-year-old woman with autism spectrum disorder and severe intellectual disability who presented with severe behavioral dysregulation and limited response to multiple psychotropic regimens. Despite progressive lithium dose escalation, serum levels remained subtherapeutic. The turning point came with the diagnosis of profound vitamin C deficiency (ascorbic acid <1 µmol/L), prompting high-dose supplementation. Within days, the patient showed rapid behavioral improvement and a marked rise in serum lithium concentrations, necessitating dose reduction. First, nutritional deficiencies—particularly scurvy—can manifest primarily through psychiatric and behavioral symptoms in vulnerable populations, often resulting in misdiagnosis and polypharmacy. Second, although still speculative, we hypothesize that vitamin C status may affect lithium pharmacokinetics. This case underscores the importance of nutritional screening in refractory psychiatric presentations, particularly in individuals with restrictive diets, and highlights a potentially underrecognized interaction between vitamin C and lithium that warrants further investigation.

## Introduction

Autism spectrum disorders (ASDs) are neurodevelopmental conditions characterized by persistent deficits in reciprocal social communication and interaction, alongside restricted, repetitive patterns of behavior and atypical sensory processing (1). Challenging behaviors are often observed in this population: self-injurious behaviors, hetero-aggression, major disruptive behaviors, or catatonia, with various grades of severity (intensity, frequency, duration, and/or localization). They can sometimes lead to severe behavioral regression, and result in dramatic and even life-threatening consequences (2). Managing challenging behavior is particularly complex in severe ASD with co-occurring intellectual developmental disorder (3, 4). Causes of challenging behavior are highly heterogeneous, including psychiatric and organic comorbidities (2, 5–7). Undiagnosed organic conditions often lead to prolonged psychotropic misuse and iatrogenic harm (8, 9).

There is scarce literature on the relationship between nutritional deficiencies and challenging behaviors in individuals with ASD. Evidence is even more limited regarding vitamin C deficiency, despite case reports of scurvy in autism (10–13) and an increasing prevalence of scurvy in recent years—particularly in the United States—where a notable proportion of cases occur in children with autism spectrum disorder (14, 15). Given that humans cannot synthesize vitamin C endogenously and rely entirely on exogenous intake, this link may be particularly relevant in the context of severe food selectivity frequently observed in ASD.

Clinically, scurvy presents with fatigue, impaired bone growth in children, and connective tissue fragility resulting in perifollicular hemorrhages, petechiae, ecchymoses, and gingival bleeding. However, its neuropsychiatric manifestations—including increased irritability, depressive symptoms, and cognitive impairment—are less widely recognized (16). Given vitamin C’s crucial role in neurotransmitter synthesis and neuroprotection, such symptoms may be underdiagnosed in individuals with developmental disorders (17) who exhibits fragility and particular sensitivity to neuropsychiatric stressors. Moreover, the widespread involvement of connective tissue in scurvy raises important questions about its hypothetical impact on drug absorption, distribution, and metabolism—particularly for medications with narrow therapeutic windows. Fortunately, treatment is simple and effective: high-dose vitamin C supplementation typically results in complete symptom resolution within days to weeks. Given the complex and pleiotropic manifestations of scurvy, clinicians should maintain a high index of suspicion in patients at risk of nutritional deficiencies—especially those with food hyperselectivity, such as individuals with ASD or intellectual disability. In this case report we describe an 18-year-old adolescent with ASD and severe intellectual disability who presents with resistant challenging behaviors requiring hospitalization in a specialist psychiatric unit dedicated to mental disability.

## Case presentation

### Developmental and psychiatric history

The patient is the second child of a non-consanguineous Portuguese couple living in Switzerland since early childhood. She has one older brother, who is in good health. There is no reported history of developmental delay within the family. Pregnancy and delivery were uneventful, although the patient was born small for gestational age (2.41 kg) by elective cesarean section at 38 weeks, with moderate neonatal adaptation (Apgar scores 5-8–9 with the requirement of a brief ventilatory support, although umbilical cord pH was normal).

The first months of life were unremarkable (No problems with feeding, sleeping or growth). Between 12 and 24 months, early milestones were delayed, with late independent sitting and walking (19 months), leading to the diagnosis of a mild global developmental delay with predominant motor impairment, but without a specific etiological diagnosis.

Between the ages of 18 months and 3 years, social engagement remained minimal. She appeared withdrawn, “in her own world,” and did not develop functional language. According to parental reports, she frequently mouthed and bit objects. At 3.5 years of age, she began guiding adults by the hand to desired objects, occasionally pointing to request items, though never for social sharing. Comprehension appeared context-dependent, with inconsistent response to her name and fluctuating understanding of spoken words. Marked behavioral reactions to frustration or opposition were noted, including screaming, hyperextension postures, hitting, and biting. Sensory observations included frequent mouthing behaviors and motor stereotypies, without tactile or auditory hypersensitivity. Interests were brief and fragmented, with constant shifting from one activity to another.

A psychological evaluation identified severe impairments in behavior, relational skills, and communication, with minimal development of pre-communication prerequisites. There were profound deficits in social interaction and significant difficulty initiating exchanges, with or without objects. Play and exploration were atypical, and object use was restricted. Based on these findings, a diagnosis of ASD with severe developmental delay was established. A multidisciplinary care plan was recommended, combining educational support for the parents at home, child psychiatry follow-up, and enrollment in a specialized school. Early investigations showed mildly enlarged ventricles on cranial CT and a non-epileptic EEG.

The clinical course was marked by persistent severe developmental delay with associated severe intellectual disability and ASD. Behavioral difficulties progressively worsened, with episodes of hetero-aggressiveness—mainly in response to frustration—and periods of self-injury, particularly self-biting.

Subsequently, she entered a non-medical residential care facility, initially, at the age of 5, part-time during the scholar hour and later, as a full-time resident at the age of 12. Although the patient is placed in a care home which represents a psychosocial stressor because of the need for the patient to adapt to the support of an educational team, she keeps in touch with her family by regularly returning to her parents’ home, where her older brother is present in her life. Her parents are supportive of her and the family bond is strong. There were no obvious traumatic events during this period of her life or lack of educationnal support. However, the non-medical’s residential care team described a gradual increase in challenging behaviors, often associated with episodes of pain or physical discomfort, or oppositional reactions to certain demands. Her medical history was marked by recurrent somatic issues, including dental malocclusion, recurrent vulvitis and urinary tract infections, gastrointestinal complaints (reflux and chronic constipation), orthopedic pain due to postural anomalies, and dietary selectivity with avoidance of fruits and most vegetables.

In 2019, a brain MRI under general anesthesia revealed hypoplasia and dysmorphic features of the corpus callosum, associated with hypoplasia of the brainstem and, to a lesser extent, the cerebellar vermis. There was no evidence of cortical gyral abnormalities or white matter signal changes. The same year, an esophagogastroduodenoscopy and coloscopy were performed under general anesthesia to evaluate the potential reflux and the chronic constipation, but no organic cause was identified.

Multiple pharmacological trials—including risperidone, escalated up to 6 mg/day with motor side effects, aripiprazole with akathisia-like side effects, as well as valproate, sedatives, and gabapentin—proved ineffective on behavioral symptoms.

Given the progressive worsening of her behavioral disturbances—arising from a complex interplay of somatic, psychiatric, and neurodevelopmental factors—and the lack of improvement despite multiple pharmacological trials, she was admitted in August 2024 to a specialized psychiatric crisis unit for individuals with intellectual disabilities.

Clozapine and lithium had not yet been trialed prior to admission. A genetic assessment was proposed based on the patient’s developmental profile but was refused by the parents.

Usual treatment:

- Gabapentine 600 mg x 3/day
- Rispéridone 2 mg x 3/day
- Mélatonine 6 mg/day at bedtime
- Macrogol 17 g 2x/day
- (R) Levomepromazine 25 mg max 4/d in case of agitation (reserve medication given only in cases of acute agitation)

### Inpatient management and clinical evolution

At admission, the patient displayed daily severe acute behavioral crisis with auto- and hetero-aggressive behaviors, constant agitation that do not respond to sedative medication. Her sleep was preserved, but she had marked food selectivity—consuming no fruits and very few vegetables—and relied heavily on transitional objects (pacifier and a plastic bowling pin).

On clinical examination, mensuration growth parameters and vital signs were normal except for mild hypertension related to stress induced by the clinical examination (Weight: 69.2 kg; Height: 160 cm; BMI: 27.03 kg/m2; Temperature: 36.9°C; BP (mmHg): 159/93; HR: 91/min; heart rhythm: regular). The neurological examination showed equinus gait with an enlarged polygon of support but no other abnormalities. The rest of the clinical examination was normal, and particularly no signs of malnutrition despite the food selectivity. The blood test on admission showed no abnormalities in renal, hepatic, pancreatic or pituitary function, no signs of infection or inflammation, no electrolytes and coagulation disorders. An initial vitamin assessment (vitamins A, B1, B6, B12, D and zinc) showed a simple folate and vitamin D deficiency wich we substituted with tablets with no other deficiencies (Vitamin C has not been measured at this stage because it is not part of the basic blood test). The rest of the metabolic assessment was unremarkable. The hepatic cytochrome P450 phenotyping test was abnormal notably, poor CYP2C9 function (evaluation of hepatic metabolic pathways) (18). The hypothesis of an obvious somatic cause explaining the clinical picture of decompensation was therefore initially ruled out.

In order to limit drug-induced iatrogenesis, particularly the effects of long-term risperidone treatment on motor function, the treatment of risperidone was switched for quetiapine leading to behavioral worsening. Treatment with quetiapine was therefore discontinued and the initial treatment with risperidone was resumed. Gabapentin was gradually tapered off due to lack of efficacy when it was introduced in the period prior to hospitalization.

During this period, the patient exhibited cyclic worsening behaviors, prompting suspicion of premenstrual dysphoria. In October 2024 continuous contraception was initiated with slight improvement. Persistent aggression, sensory dysregulation, and potential migraine equivalents (facial grimacing, withdrawal to dark areas, facial self-hitting) led to trials of triptans without benefit. In order to contain the agitation, trials of various sedatives (chlorpromazine, levomepromazine, lorazepam, clonidine) failed, with clonidine causing a grade II anaphylactic reaction.

In November 2024, catatonia was hypothesied due to bizarre posturing, repetitive motor behaviors, mutism, food refusal, and disorganized behavior. A lorazepam challenge (up to 12 mg/day) failed. Due to the dangerous behavior, she was placed in psychiatric intensive care isolation.

Subsequently, clozapine was introduced due to the failure of previous attempts with antipsychotics with gradual titration to 375 mg/day with the aim of gradually discontinuing risperidone once the clinical condition has stabilized. The patient developed tachycardia up to 120 bpm managed with propranolol. At the same time, given a picture combining mood swings with rapid shifts between periods during which the patient exhibited asthenia, loss of acquired skills and carelessness, and periods during which she appeared agitated, euphoric, irritable and aggressive, the hypothesis of a rapid-cycling bipolar mood disorder was made and lithium treatment was introduced, though initial serum levels remained subtherapeutic despite doses up to 38 mmol/day. Although only partially effective, these therapeutic adjustments, combined with intensive care provided in the isolation room, resulted in partial clinical improvement. This allowed, from first March, a gradual reintegration into daily activities in the communal area with extended periods in isolation rooms, both fixed during the day and in response to episodes of agitation.

Mid-March 2025, the patient developed spontaneous gingival bleeding without any other specific signs. Given her dietary habits, scurvy was suspected and confirmed with undetectable vitamin C levels (<1 µmol/L). High-dose vitamin C (1g/day) was initiated.

**Figure 1:**
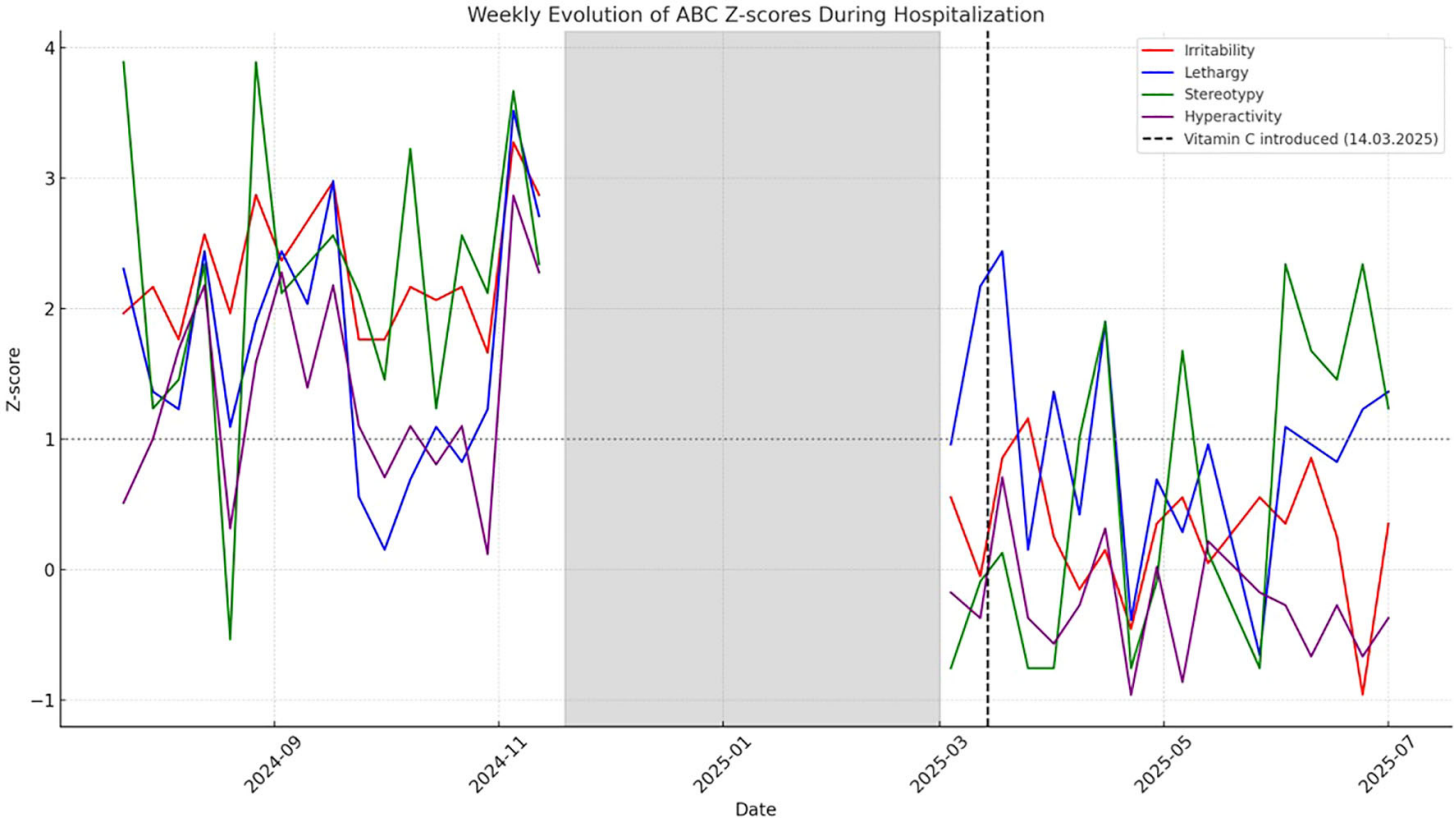
Evolution of ABC scores—comparison with a population with severe intellectual disability.

Following vitamin C supplementation, a rapid and sustained clinical improvement occurred: reduction in aggressiveness, enhanced emotional regulation, greater capacity for non-verbal communication, and a previously unseen ability to accept frustration and engage in social interactions. She engaged more with caregivers and peers and was receptive to structured activities. This clear and rapid improvement alloweds for a complete end to isolation, with clinical stability persisting despite the resumption of group life. **Figure 1**. shows the weekly evolution of the patient’s Aberrant Behavior Checklist scores during hospitalization (ABC is a well-established assessment tool for challenging behaviors in people with intellectual disabilities and autistic people.) (19). Scores above the Z-score baseline of 1.0 are considered significant relative to the population with severe intellectual disability (SID). From 19th November 2024 to 1st March 2025, the patient was placed in psychiatric intensive care isolation due to dangerous behavior and could not be assessed behaviorally. Between the 1st March and 1st April, the patient benefited from a gradual easing of hospital restrictions and was reintegrated into group life. As of 1st April, the patient was no longer being treated in an isolation room. Although we observed an improvement in ABC scores from 1st March onwards, it is difficult to interpret the data between 1st March and 1st April due to the prolonged period of isolation in isolation rooms. Vitamin C treatment was initiated on 14th March 2025, indicated by the vertical dotted line.

**Figure 2:**
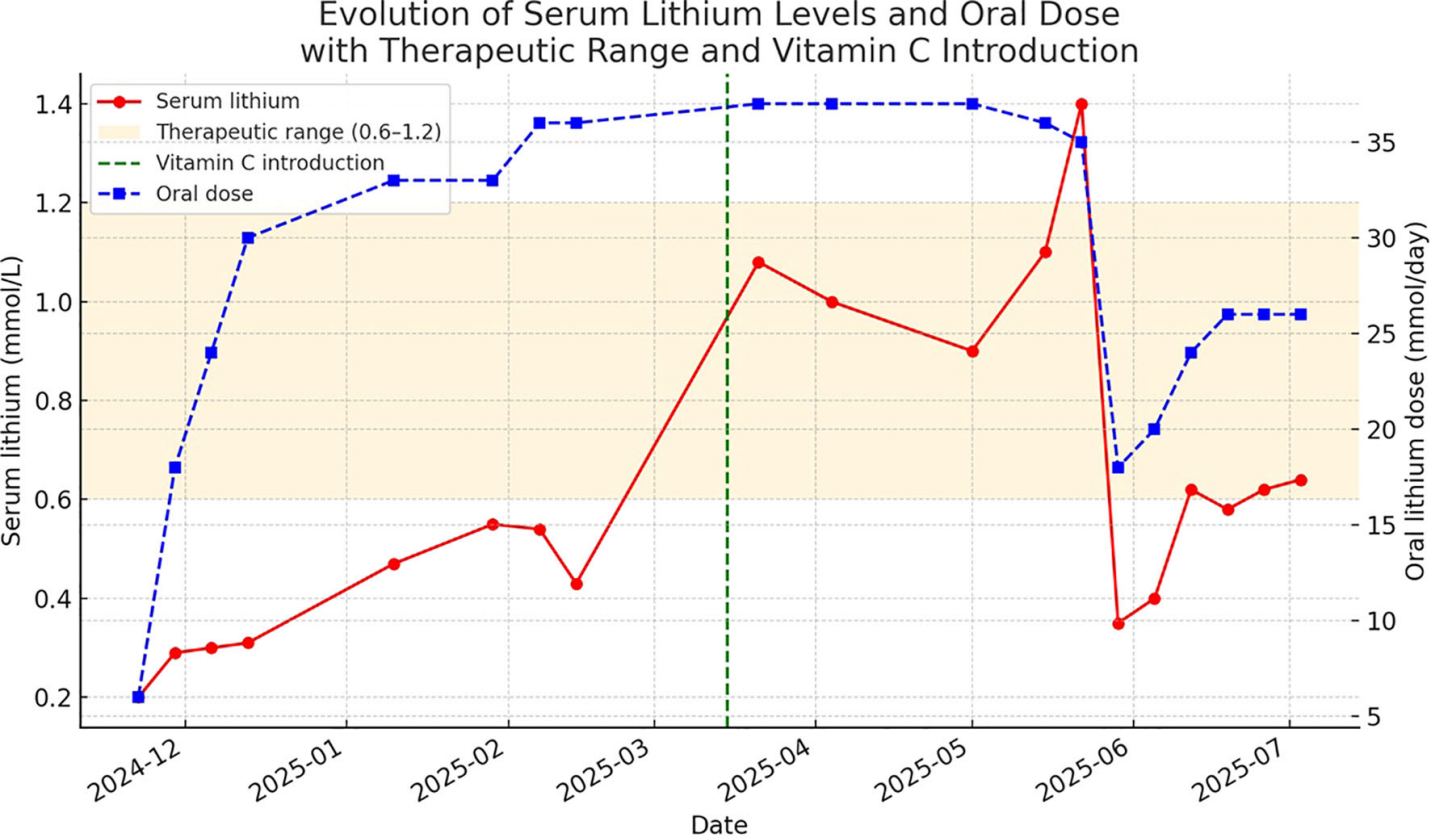
Evolution of serum lithium and oral dose before and after vitamin C supplementation.

Notably, lithium serum levels surged from subtherapeutic to a range between 0.8 and 1.1 mmol/L with a peak of 1.4 mmol/L, prompting immediate dose halving and stabilization at 25 mmol/day with therapeutic levels (~0.6 mmol/L). **Figure 2** presents the evolution of serum lithium levels and oral lithium dose (in mmol/L and mmol/day) before and after the initiation of vitamin C supplementation on 14th March 2025. The graphic illustrates a marked increase in the serum level following vitamin C administration, suggesting improved lithium absorption or decreased elimination. Following the introduction of vitamin C supplementation, the mean serum lithium-to-oral dose ratio increased from 0.0165 to 0.0264, representing an approximate 60% relative increase. This supports the hypothesis that correction of scurvy may influence pharmacokinetics, potentially through restoration of intestinal or renal transport mechanisms. It should be noted that medication adherence was good throughout the hospitalization period, with no fluctuations.

This behavioral and metabolic stabilization permitted tapering of lorazepam and risperidone; the latter reduced to 1 mg/day by May 2025. The patient was transitioned to a new residential care setting with sustained improvement and support for intensive psychoeducational interventions.

Her treatment upon discharge was: Clozapine 350 mg/day for irritability in a patient with severe ASD who had developed severe side effects with two other antipsychotics since childhood, Inderal 120 mg/day as a treatment of tachycardia induced by clozapine and a second-line treatment for anxiety, lithium 26 mmol/day for suspected bipolar mood disorder, Risperidone 1 mg/day with the aim of gradually discontinuing it completely and replacing it with clozapine, folate, vitamin D and vitamin C supplementation due to the aforementioned deficiencies and the patient’s selective eating habits, Fosfomycin 3g/week to prevent recurrence of urinary tract infection.

The six-month follow-up after discharge from the hospital confirms clinical stability: the patient lives in a two-resident facility and participates in daily life without behavioral crises.

## Discussion

One hypothesis for the positive clinical outcome could be that the concurrent adaptation of psychoeducational care and educational management, combined with the low level of stimulation to which the patient is exposed in the psychiatric intensive care isolation room, as well as clozapine and lithium treatment, which acts both on behavioral disorders resistant to other treatments and on bipolar-type mood disorders. However, blood lithium levels remained low until treatment with vitamin C, suggesting a lesser therapeutic effect, and clinical improvement was very slow before March 2025. It was only after the introduction of vitamin C supplementation that the patient was able to fully reintegrate into the unit’s social life without long-term use of the psychiatric isolation room and showed a clear and rapid improvement in baseline behavior as well as good self-regulation skills during periods of frustration (with spontaneous use of certain sensory strategies such as rocking or putting objects or hands in her mouth), which was a new behavior and therefore suggests a clear decrease in the patient’s overall level of irritability.

Another hypothesis could have been the gradual resolution of a somatic problem other than scurvy, such as inflammation, chronic infection or gastrointestinal problems, which would have led to a gradual reduction in the patient’s general discomfort and allowed more appropriate behavior to emerge. However, the laboratory tests carried out during this period showed no sign of inflammation, no renal or hepatic dysfunction and no dysregulation of electrolytes. The patient, known to have recurrent urinary tract infections, probably due to chronic carriage of E. Coli in her urine, was receiving preventive treatment with Fosfomycin once a week with good clinical results. A gastroscopy and colonoscopy were performed in the summer of 2019 and showed no abnormalities, and hormone treatment was introduced based on the hypothesis of premenstrual syndrome, as mentioned above. All common somatic causes had therefore been ruled out.

Although scurvy is a condition classically associated with physical symptoms such as gingival bleeding, poor wound healing, and fatigue, its neuropsychiatric manifestations are increasingly recognized, particularly in populations with cognitive or developmental disabilities (16, 20). In this case, an adolescent with autism and severe intellectual disability exhibited a complex clinical picture: chronic irritability, frequent aggression, stereotyped behaviors, and intense resistance to care. These symptoms were originally attributed to her underlying neurodevelopmental condition and psychosocial stressors (placement in a non-medical care home with support mainly from educators), leading to numerous psychopharmacological interventions with limited success.

The diagnosis of scurvy emerged only after a spontaneous gingival hemorrhage, a classic but often late manifestation (21). Given her near-complete avoidance of fruits and vegetables, severe vitamin C deficiency was biologically confirmed. Remarkably, within days of initiating high-dose vitamin C supplementation, the patient exhibited dramatic behavioral improvements, as mentioned above.

This clear clinical improvement was the result of multidisciplinary care (medical, educational, psychological and pharmacological). Given the various treatments and psycho-educational approaches tried at the same time, it is difficult to attribute the success of the therapy to a single cause, which is the main limitation of this study. However, the rapid transformation strongly suggests that the scorbutic state had significantly exacerbated her psychiatric profile. Vitamin C is essential for the biosynthesis of dopamine, norepinephrine, and serotonin, as well as for modulating oxidative stress and neuroinflammation—mechanisms increasingly implicated in psychiatric symptoms (16, 17, 20, 21). In patients with autism and cognitive impairment, where communication barriers hinder symptom expression, scurvy may manifest predominantly as behavioral dysregulation or agitation. Misattributing such symptoms solely to psychiatric illness may delay appropriate nutritional diagnosis and treatment. This raises the question of the long-term maintenance of the rest of the medication, particularly Clozapine, Inderal and Lithium, which can cause long-term side effects. A gradual withdrawal of the remaining medications should be considered once the patient is well stabilized in her outpatient setting. An unexpected and clinically significant observation in this case was the sharp increase in serum lithium levels following vitamin C supplementation. Despite progressively increasing lithium doses up to 38 mmol/day, the patient had persistently low serum levels prior to vitamin C repletion. Within days of initiating vitamin C therapy, lithium levels rose from subtherapeutic to a range between 0.8 and 1.1 mmol/L—necessitating drug discontinuation, then reintroduction at half the prior dose.

Lithium is freely filtered at the glomeruli, reabsorbed mainly in the proximal tubule (80%) but also to a lesser extent in the collecting duct via the sodium channel (ENaC), located at the apical membrane of the principal cells. Lithium is eliminated exclusively by the kidneys. Lithium levels are directly influenced by glomerular filtration rate (GFR) and the degree of tubular reabsorption. In the event of a decrease in GFR (renal failure, NSAIDs, ACE inhibitors) or increased tubular reabsorption (low-salt diet, hypovolemia of any origin, including when taking diuretics), blood lithium levels will increase. Conversely, an increase in glomerular filtration or a decrease in tubular reabsorption leads to a decrease in blood levels (22). This occurs either during co-prescription with drugs that induce lithium hyperfiltration in the kidneys: osmotic diuretics, carbonic anhydrase inhibitors, carbamide, calcitonin, SGLT-2 inhibitors, alkalising substances or substances containing large amounts of sodium, topiramate, xanthines (caffeine, theobromine, theophylline), which was not the case in this patient, or a medical condition that could affect kidney function. The blood tests carried out during this period returned to normal, particularly fasting blood glucose, sodium levels and kidney function, which ruled out any medical condition of this type.

The temporal association between the introduction of vitamin C treatment and increased blood lithium levels raises the question of whether scurvy affects lithium pharmacokinetics and more broadly other renal elimination molecules sensitive to the absorption/reabsorption mechanism. Although the only well-established pharmacokinetic interaction of ascorbic acid involves psychostimulants—where urinary acidification increases amphetamine clearance and reduces clinical efficacy (23)—evidence concerning antipsychotics and antidepressants remains limited. However, no consistent effects on serum drug concentrations have been demonstrated, and no direct causal mechanism has been established in the literature regarding interactions with lithium or other renally eliminated agents. Several hypotheses may be considered:

• Urine Acidification: The combination of vitamin C and lithium could increase serum lithium concentration through urine acidification, which would reduce renal excretion (24). However, although this interaction is mentioned on the Epocrates drug interaction website (25) without any available sources, there are no references in the literature confirming this interaction.

• Renal Reabsorption: Lithium is primarily excreted through the kidneys. Vitamin C plays a role in maintaining renal tubular function and may influence sodium and lithium transporters. In vitamin C deficiency, altered proximal tubular reabsorption could hypothetically impair lithium reuptake, increasing renal clearance (26).

• Oxidative Stress and Renal Transporters: Vitamin C’s role as an antioxidant may affect the expression of renal transporters. Chronic deficiency could lead to suboptimal function of sodium-lithium counter-transport mechanisms, indirectly affecting serum levels (27).

• Gastrointestinal Absorption: Although no direct evidence confirms that vitamin C deficiency alters gastric pH or intestinal permeability, it is biologically plausible that chronic ascorbic acid depletion may contribute to subtle mucosal dysfunction or altered gastrointestinal environment, which could, in turn, affect the solubility or absorption of orally administered compounds such as lithium (28).

While the precise mechanism remains unclear, the pharmacokinetic shift observed here was both abrupt and clinically relevant. This suggests that scurvy may subtly alter the pharmacokinetics of lithium, an observation deserving of further study—especially in populations where both conditions (mood instability and poor nutrition) may coexist.

Clinicians managing lithium therapy in patients with malnutrition, restrictive eating patterns, or unexplained serum level variability should consider vitamin C status as a potential modifying factor. Monitoring for nutritional deficiencies may improve not only clinical outcomes but also the safety and predictability of pharmacological interventions.

This hypothesis remains purely speculative given the lack of data on this subject in the literature and this represents the second main limitation of this study. However, it warrants further investigation as the pharmacokinetics of other drugs could also be affected by serum vitamin C levels.

## Conclusion

This case illustrates the profound clinical impact of an unrecognized vitamin C deficiency in a young woman with autism and severe intellectual disability, presenting with severe behavioral dysregulation and apparent pharmacoresistance. The identification and correction of scurvy led to rapid psychiatric stabilization, allowing simplification of the pharmacological regimen and enhanced psychosocial functioning. Scurvy, though rare, remains a relevant and reversible contributor to psychiatric decompensation and pharmacological unpredictability. Early recognition may prevent misdiagnosis, reduce polypharmacy, and significantly improve patient outcomes. Systematic vitamin C screening in all psychiatric patients may be unrealistic; however, the presence of the red flags specified in **Table 1** should prompt clinicians to consider measuring vitamin C levels or initiating supplementation, particularly in high-risk populations such as individuals with autism and severe food selectivity.

**Table 1:** Red flags indicating that vitamin C blood measuring may be advisable.

Domain	Red flags
Neurodevelopmental context	Autism spectrum disorder, severe intellectual disability, restrictive/ARFID-like eating patterns
Behavioral presentation	Treatment-resistant challenging behaviors (aggression, irritability, self-injury)
Dietary history	Extreme food selectivity, absence of fruits and most vegetables
Oral/dental signs	Gingival bleeding, gum swelling, poor wound healing
Musculoskeletal signs	Arthralgia, myalgia, impaired mobility, painful gait, delayed bone healing
Dermatologic signs	Petechiae, ecchymoses, perifollicular hemorrhages, hyperkeratosis
General symptoms	Fatigue, loss of appetite, mood changes, unexplained regression

Additionally, a striking increase in lithium serum levels post-treatment highlighted a possible influence of vitamin C on lithium pharmacokinetics.

Consent for publication was obtained from the patient’s legal guardian.

## Data Availability

All the patient's medical data used for this case report comes from her secure medical file held by the CHUV.
